# Untangling Classification Methods for Melanoma Skin Cancer

**DOI:** 10.3389/fdata.2022.848614

**Published:** 2022-03-29

**Authors:** Ayushi Kumar, Avimanyou Vatsa

**Affiliations:** ^1^Monroe Township High School, Monroe Township, NJ, United States; ^2^Department of Computer Science, Fairleigh Dickinson University, Teaneck, NJ, United States

**Keywords:** melanoma, skin cancer, classification, CNN, RNN, XG-boost, performance

## Abstract

Skin cancer is the most common cancer in the USA, and it is a leading cause of death worldwide. Every year, more than five million patients are newly diagnosed in the USA. The deadliest and most serious form of skin cancer is called melanoma. Skin cancer can affect anyone, regardless of skin color, race, gender, and age. The diagnosis of melanoma has been done by visual examination and manual techniques by skilled doctors. It is a time-consuming process and highly prone to error. The skin images captured by dermoscopy eliminate the surface reflection of skin and give a better visualization of deeper levels of the skin. However, the existence of many artifacts and noise such as hair, veins, and water residue make the lesion images very complex. Due to the complexity of images, the border detection, feature extraction, and classification process are challenging. Without a proper mechanism, it is hard to identify and predict melanoma at an early stage. Therefore, there is a need to provide precise details, identify early skin cancer, and classify skin cancer with appropriate sensitivity and precision. This article aims to review and analyze two deep neural network-based classification algorithms (convolutional neural network, CNN; recurrent neural network, RNN) and a decision tree-based algorithm (XG-Boost) on skin lesion images (ISIC dataset) and find which of these provides the best classification performance metric. Also, the performance of algorithms is compared using six different metrics—loss, accuracy, precision, recall, F1 score, and ROC.

## 1. Introduction

In the United States, skin cancer is the most common form of cancer. In particular, melanoma is the most common and specific form of skin cancer that is malignant. It starts when melanocyte cells (which give skin the tan color) begin to overgrow, and skin color changes darker. Melanoma may occur on normal skin or may appear as a mole or other region of the skin experiencing changes. A few moles can turn into melanoma when they emerge during childbirth. Cancer cells begin to grow because the skin cells' DNA is damaged from ultraviolet rays, and that causes mutations or triggers genetic mutations. This damage leads to multiplying of skin cells multiplying or growing at a much faster pace and causing malignant tumors also grow (Milton, 2018; Poongodi et al., 2021; Sharma et al., 2021).

Melanoma type of cancer may spread to the other parts of the body (eyes, ears, gingival of the upper jaw, tongue, lips, etc.) if it is not treated as soon as identified (early stage). Melanoma spots can develop anywhere on someone's body, but for men, they are common on the chest and back, and then for women, it is more common on the legs (Milton, 2018). The prevalence of melanoma has dramatically increased in the past 50 years, and the incidence of melanoma has risen by 4–6% in predominantly fair-skinned populations in North America, New Zealand, Australia, and Northern Europe (Kosary et al., 2014). Melanoma may be characterized by its common phenotypes like multi-color (usually a combination of a few hues). It has an unpredictable asymmetrical shape (cannot be divided in half) and is intermittent. Also, its width is more prominent than 6 mm in diameter, and it may enlarge or develop moles that eventually change shape and size and will typically advance toward being a melanoma (Gurung and Gao, 2020).

**Figure 1:**
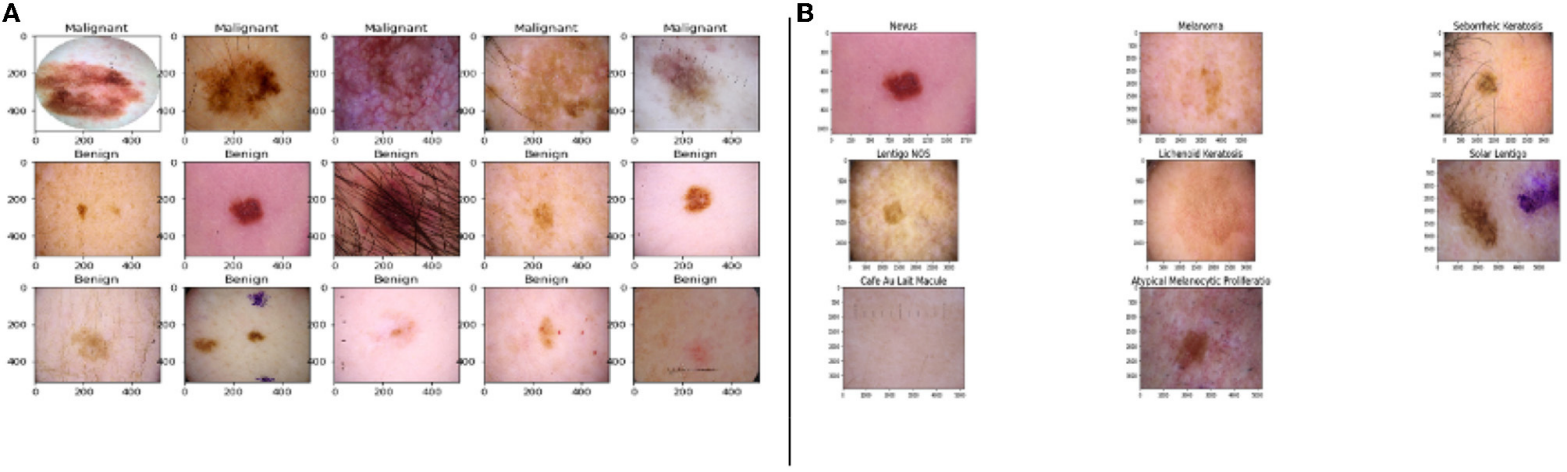
Images from the dataset, **(A)** samples of benign and malignant images, **(B)** the five major stages of skin cancer (Esteva et al., 2017; Haenssle et al., 2018).

The images listed in Figure 1A shows the malignant and benign (non-cancerous images) lesions.

The primary five skin lesion groups constitute about 90% of the lesions contained in routine skin examination: actinic keratosis [intraepithelial carcinoma / Bowen's disease (Class A)], basal cell carcinoma (Class B), benign keratosis including seborrheic keratosis, solar lentigo, and lichen planus-like keratosis (Class K), melanocytic nevi (Class N), and melanoma (Class M) (Esteva et al., 2017; Haenssle et al., 2018).

Figure 1B shows the sample of eight phenotypes - Nevus, Melanoma, Seborrheic Keratosis, Lentigos, Lichenoid Keratosis, Solar Lentigo, Cafe Au Lait Macules, and Atypical Melanocytic Proliferation. Nevus is generally used to describe benign clusters of skin cells or moles. Lentigo NOS, also known as Nevoid Lentigo, is a benign skin lesion that can appear on any part of the body. Melanoma is the most dangerous form of skin cancer. Seborrheic Keratosis is also a benign skin growth. A Lichenoid Keratosis is a benign macula that can appear on the skin. Solar Lentigo, also known as liver spots, is a Lentigo that occurs due to excess exposure to the sun. Cafe au Lait Macules are common birthmarks that present as hyperpigmented skin patches with defined borders. Atypical Melanocytic Proliferation is histopathological samples with no defined diagnosis (Esteva et al., 2017; Haenssle et al., 2018; Sahu et al., 2018, 2019).

Melanoma can be easily treated by resecting the skin lesion if it has been caught in an early stage. However, in a later stage, melanoma becomes more challenging to treat. Therefore, self-examination and early diagnosis are crucial in the effective treatment of melanoma (Voss et al., 2015). However, the confirmation of melanoma is done by images that have been taken by dermoscopy. For dermatologists, the sensitivity rate in the unaided diagnosis of dermoscopic images is less than 80% (Psaty and Halpern, 2009). Thus, computer-aided diagnosis of melanoma provides promise in increasing the sensitivity of melanoma diagnosis.

In the USA, melanoma represents a small portion (~1%) of annual skin cancer diagnoses but accounts for more than 65% of the deaths attributed to skin cancer (Cancer-Society, 2021; WHO-Cancer, 2021). As described in an observational study, the melanoma growth rate increases at a rate of 0.13 ± 0.16 mm/month (Betti et al., 2016). In turn, at depths greater than 0.3 mm, melanoma is at risk of metastasizing. However, melanomas can be treated on early detection, which is the optimal strategy (Betti et al., 2016).

Magnetic resonance imaging (MRI) or nuclear magnetic resonance (NMR) imaging is also a popular method to find cancer in the body. In addition, it helps doctors to plan cancer treatments such as surgery or radiation. MRI takes cross-section images by using strong magnets – not radiation. It scans the cross-sectional image from many angles to understand the insight of the image. However, MRI is primarily suitable for brain and spinal cord tumors or other soft tissue parts of the body (Cancer-Society, 2021; WHO-Cancer, 2021).

The first phase in a dermatologist's diagnosis of a malignant lesion is a visual inspection of a suspicious skin area. A correct diagnosis is critical because of the similarity of certain types of lesions; besides, diagnostic precision is strongly linked to the doctor's expert experience. This will benefit both physicians and non-experts by saving time by automating the first diagnostic phase. Thus, the implementation of computer-aided diagnosis seeks to assist healthcare providers in allowing for more frequent screening with better diagnoses. After that, the classical machine learning workflow was used, which includes preprocessing, segmentation, extraction of data, and classification of images.

Nonetheless, the classification process of the dermoscopy images is still confusing because of the presence of artifacts, noises, the complexity, and the changeability of the skin lesion structures. Also, the variation of illumination during the image capturing process, dense hairs, air bubbles, and a low level of contrast between the normal skin and lesion, that cannot meet the requirements of the lesions' border detection, feature extraction, and classification processes (Codella et al., 2018; Tschandl et al., 2018; Guo and Ashour, 2019). It has been observed that if even a single pixel of melanoma was present in non-malignant lesions, the whole lesion was classified into melanoma. Even spectral distributions of lesions are overlapping to each other, so explicit mastery of an abnormal state of use is required, particularly for extraction of features, and the choice of satisfactory feature is very tedious (Pölönen et al., 2019; Ratul et al., 2019).

Moreover, in order to classify skin images among benign or malignant or other types of skin cancer, the skin images are used for accurate testing and predicting by trained machine (Moolayil, 2021; Saha, 2021). Therefore, data-centric-based deep learning methods may detect skin cancer accurately and in the early stages and delineate them properly from healthy tissue. These methods use numerous layers of nonlinear data processing to extract, recognize patterns, and characterize features. A model in deep learning describes how to characterize from pictures, text, or sound. Similarly, when a machine is prepared to use significant quantities of information collections, and after that, the pixel estimate of a picture is modified to an internal portrait or vector highlight where classifiers may identify or group design in the information. Therefore, there is a need to untangle the deep learning-based classification methods (convolutional neural network, CNN; recurrent neural network, RNN; and XG-Boost) for skin lesions. The classification methods facilitate two major help to provide precise details and treatment options for a lesion and to identify early skin cancer with appropriate sensitivity and precision (Esteva et al., 2017; Haenssle et al., 2018).

In this article, the experiment was carried out by determining many networks, optimization methods, and an appropriate number of epochs to measure six different performance parameters – loss, accuracy, precision, recall, F1 score, and ROC. These performance parameters are compared among three algorithms – CNN, RNN, and XG-Boost. Also, all models resulted from the training by 28,157 (total) dermoscopic images and were tested with 4,969 images.

The rest of this article is organized as follows. This section explains the introduction, and Section 2 includes the background and related work. The material and methods used are included in Section 3, whereas Section 4 describes the result and discussion based on the considered dataset. Finally, Section 5 includes a conclusion.

## 2. Background

In recent years, the rise of Deep Neural Networks (DNN) and their application have been seen in various fields, including medical image classification. The DNN provides a promising method for rapid melanoma identification and classification. Consequently, many researchers have applied some deep learning-based classification methods - CNN, RNN, and XG-Boost - for medical image classification (Codella et al., 2018; Tschandl et al., 2018; Guo and Ashour, 2019; Moolayil, 2021; Saha, 2021). These algorithms are coupled with increased algorithmic sophistication and computing power. These algorithms make melanoma skin cancer detection better since the deep learning algorithms leverage biological structure and a data-centric decision approach (Lakshmanaprabu et al., 2018; Swain et al., 2020).

The convolutional neural network is a derivative of a feedforward neural network and uses a series of convolutional, pooling, and fully connected layers. These layers find the main features from the images to be accurately classified. The convolution layer uses a convolutional product using the vertical edge filter. However, the edges' information of images is thrown away since the pixels on the corner of the image are not much-used w.r.to. the pixels in the middle of the picture. Thus, CNN adds zeros as the padding bits around the image. The convolutional product between tensor and filter may be defined after the definition of stride (to shrink the size of output and vice-versa) and padding. The pooling layer down samples the image's features by summing up the information. This operation needs to be carried out through each channel. We may apply either the average pooling or max-pooling process (Corizzo et al., 2021; ÜNLÜ and ÇINAR, 2021).

Additionally, the CNN is a Multilayer Perceptron (MLP) advancement, and it is designed to process two-dimensional information. Because of the high network density and many applications to image information, CNN integrated into DNN. Also, CNN uses images to learn functions to differentiate from one pattern to another. The pre-processing steps for CNN are more manageable, and there are fewer steps than other types of classification algorithms (Saha, 2021).

Convolutional neural network also shows great success in different pattern-finding tasks, including classifying melanomas and other skin cancers from dermatoscopy and regular color images. In order to transfer the learning, most of the models are initialized based on a pre-trained CNN mode like VGG16, Densenet121, Xception, InceptionV3, EfficentNetB0, ResNet50V2, and Custom. CNN's first stage, the training dataset, is used to train a model with an appropriate number of iterations. The second stage identifies the samples whose classification results have poor performance for further use to train the next model repeatedly. All procedures will be finished iteratively when they are all trained. Next, the results from the first two stages are going to be tested to evaluate the performance score of different models. Finally, the samples are predicted by using the trained models with the high-performance score (Pölönen et al., 2019; Ratul et al., 2019).

Moreover, an experiment was carried out on the RNN algorithm. It is derived from feed-forward networks and works similarly to the human brain. Also, it helps in predictive results on sequential data since RNN can remember essential information about the input they received. Therefore, it is also known as a feed-forward-based neural network where information moves in only one direction: from the input layer, through the hidden layers, to the output layer. It also acts based on current inputs and it has not much memory about the past except training. Additionally, RNN cycles information through a loop. It copies the outputs, loops them back into the network, and finally decides. Its final decisions are based on current inputs and whatever it learned from input in the past. Therefore, we may say that RNN adds its immediate past to the present in its decision.

Finally, an experiment was also carried out on XG-Boost. It is an efficient, gradient-boosting-based tree algorithm. The training was iterative by introducing new trees that predicted the previous tree's error, which was then connected to the earlier trees to generate a final classification. This article includes the result of XGBoost's models for each image set to get the broadest possible overall test range. The XGBoost algorithm's hyperparameters were balanced by randomized search. The “RandomizedSearchCV” function available in sci-kit learn was used for this purpose which uses cross-validation. Therefore, the respective test set was not used to optimize the hyperparameter of the algorithm (Esteva et al., 2017; Haenssle et al., 2018; Milton, 2018). XG-Boost learned the relationship between the true class mark, the aforementioned CNN probability distributions, and the dermatologists (Liu et al., 2019; Petrovska et al., 2021).

## 3. Materials and Methods

Every year, International Skin Imaging Collaboration (ISIC) announces challenges and an annual competition geared toward developing new algorithmic techniques for the detection of skin cancer. In this experiment, the images were used from the public repository of ISIC (ISIC-Archive, 2021). We considered 33, 061 dermoscopic images to measure the performance parameters of three algorithms – CNN, RNN, and XG-Boost. The descriptive statistics of this dataset are illustrated in the next section. We created models against which we ran our datasets for each of these algorithms – training, test, and validation. Each of these models was based on a different underlying architecture with its pre-trained base model. To these base models, we added specific individual layers to further fine-tune the results. Also, the loss values and learning rate have been optimized by varying the number of epochs.

The CNN models are initialized by seven CNN architectures, VGG16, Densenet121, Xception, InceptionV3, EfficentNetB0, ResNet50V2, and Custom, in order that the transfer learning rate may maximize. Also, we tested three different kinds of feature learning structures, 1D, 2D, and 3D convolutions. We used Stochastic Gradient Descent (SGD) based optimization to reach the lowest cost value faster than other optimizers. In this process, the classifier model is trained by an appropriate number of epochs for each image. We also guaranteed that the training data set does not include data samples from the image, which are currently under test and validation dataset (Pölönen et al., 2019; Ratul et al., 2019). After that, we processed the following four stages. The first network is trained using all training set for limited iterations to speed up training. The second stage is also in the training process, identifying the samples whose classification results have poor performance to train the next model repeatedly. All procedures will be finished iteratively when they are all trained. Next, the results from the above stage are going to be tested to evaluate the performance score of different models. Finally, predict the samples using satisfied models with a high-performance score.

We use VGG16 as the base model for the classifier. VGG16 has good scores with a 0.9 accuracy when tested on ImageNet and a fast prediction time of 4 ms along with 138, 357, 544 parameters. We resize our images to uniform 128 * 128 pixels. We are using the pre-trained weights from ImageNet to get a good starting point prior to the training. We use dense and pooling layers with the activation functions: rectified linear unit (ReLU) and sigmoid. After that, we use the SGD optimizer to control the learning rate. For the top layers, we use the default, but we later switch to 0.0001, which is much lower for the remaining layers of the model. We use binary cross-entropy loss as we only have benign or malignant classes. We also have used the early stopping callback, which monitors the F1 score every 50 epochs to ensure the model is still learning and improving. Lastly, we train the model using these parameters and evaluate them for results using the Keras model.fit() and model.evaluate() functions.

The RNN models' architectures are initialized by Bidirectional, GRU, LSTM, and SimpleRNN networks. The images fed to these models use three channels and are 128 * 128 pixels. We use the pre-trained weight from ImageNet for transfer learning of these base models. Various layers like pooling and dense layers are then added to the base model. Activation functions used for these layers are primarily ReLU and Sigmoid. For the model, we use the SGD optimizer with the default learning rate for the initial training of the top layers in our first iteration. The model has then trained again with a much lower learning rate (0.0001) for the remaining layers of the model. We use the binary cross-entropy as our loss function since we have only two classes in our problem. Finally, the model is fit by providing a class weight to manage the data imbalance between the two classes. The weight corresponds to the ratio of the instances of both the classes in the given dataset.

The XG-Boost models' setup is slightly different. Images were initially fed to a CNN VGG16 model but only extracted the features. We simply used the pre-trained weights for the purpose without actually going through the VGG16 training. The predicted features from this model were then reshaped to fit as an input to the XGB classifier model. The XGB classifier we used as a booster was gbtree, which is meant for classification. The default learning rate was 0.3 here but was reduced to 0.1 to get better results. This uses cross-entropy as the loss function. Here, too, we used class weight (scale_pos_weight) to manage the huge data imbalance. We trained the models for 150 epochs in this case.

### 3.1. Experiment and Dataset

The dataset used for this article consists primarily of images of skin lesions that could potentially be melanoma. It was generated by the ISIC, which gathered these images from various hospitals worldwide. The images were provided in multiple formats (DICOM, JPEG, and TFRecord). The training data consisted of 33,061 images and had all the attributes for each image, including the classification. There were also 10,000+ images without the actual classification, but we did not use that. Instead, we split the training data into three sets – 70% for training, 15% for validation, and the remaining 15% for testing.

Moreover, the images also came with a bunch of metadata which included the age, sex, and the location of the lesion. Another interesting bit about this data is that multiple images from the same person. All these images come from less than 2,000 individuals. While we have not leveraged this data yet, we plan to incorporate them in the next iteration to have better prediction with these additional features.

The experiments ran and tested on Kaggle, which is a customizable GPU processing environment, and it uses Jupiter notebook (ISIC, 2021). In order to set up the experimental environment on Kaggle, we used a high-level API of TensorFlow called Keras framework (Abadi, 2015; Chollet et al., 2015). Also, we imported packages from Keras, TensorFlow, NumPy, Pandas, Scikit-learn, etc., for data preprocessing and evaluating these models. Before building the model, images from the ISIC dataset are to first be preprocessed. Specifically, the images will be turned into NumPy arrays using their Red, Green, and Blue (RGB) color intensity values. Due to resizing them before the implementation, the NumPy array should be created without issues. Labels for the images were created, merged, and shuffled. Finally, the labels were turned into one-hot encoding, and the images were normalized by linear normalization.

In the CNN model building process, the Keras Sequential API will be used where one layer is added at a time, starting from the input. Multiple layers will be used in this model building process: Conv2D layer, MaxPool2D layer, combination layer, flatten layer, and fully-connected (Dense) layer. Finally, the model will be cross-validated and tested (Milton, 2018).

Recurrent neural network model will also be based on the Sequential model, where different types of RNN layers will be added to the base model. We will leverage LSTM, GRU, SimpleRNN, and bidirectional RNN layers surrounded by the input and output layers.

For the XGBoost, we will start with a pre-trained VGG16 model. This will be used to predict our images, and those predictions will be used as input of the actual XGBoost model. XGBClassifier will act as a base for this model.

### 3.2. Labeling of Images

The images were preprocessed before they were fed to the various models. Since we used TensorFlow/Keras API framework, we need to arrange and split the training images based on their classification such that each image can be tagged with the correct label while loading into Keras. Once loaded, these images were converted to floating-point values so as to provide greater accuracy and wide range, although it increases the memory requirement. After that, the dataset is split into three sets - training, validation, and testing, as mentioned earlier.

## 4. Results and Discussion

**Figure 2:**
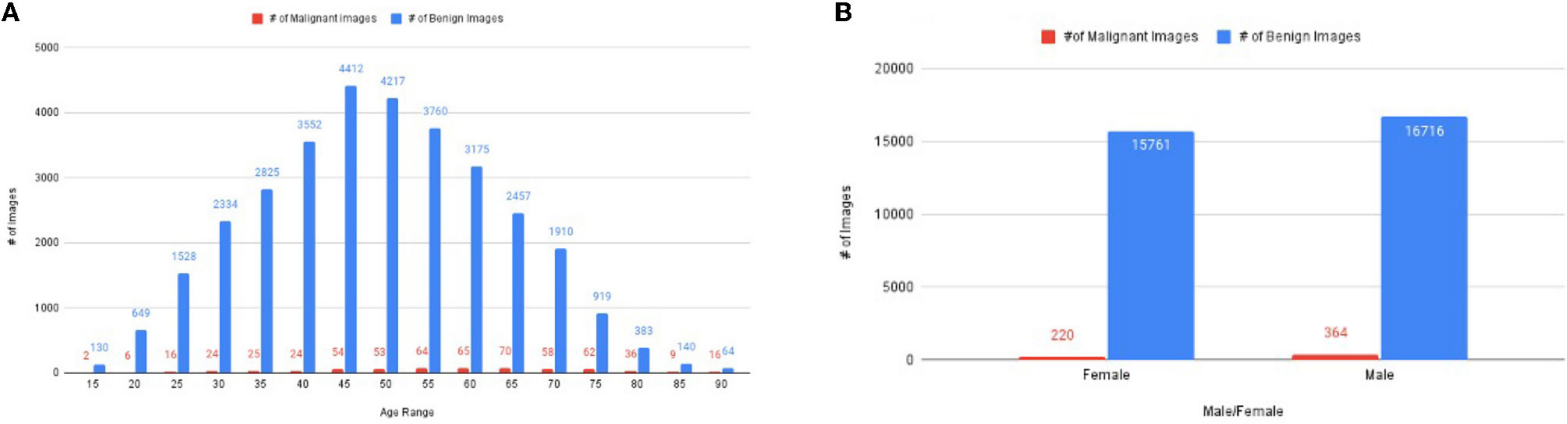
The distribution of images, **(A)** number of images corresponding to age, **(B)** the number of images of malignant and benign corresponding to gender.

The descriptive statistics of dataset w.r.t gender and age corresponding to a number of malignant and benign images are shown in Figure 2.

The dataset has 33,061 diverse groups of images, which has an age range between 15 years to 90 years (Figure 2A). It has 220 malignant images of women and 364 malignant images of men (Figure 2B). The rest of the images are benign images of men (15,761 images) and women (16,716 images) (Figure 2B). Figure 2A illustrates the number of malignant images most in the age between 25 and 80 years.

### 4.1. Outcome of CNN

**Figure 3:**
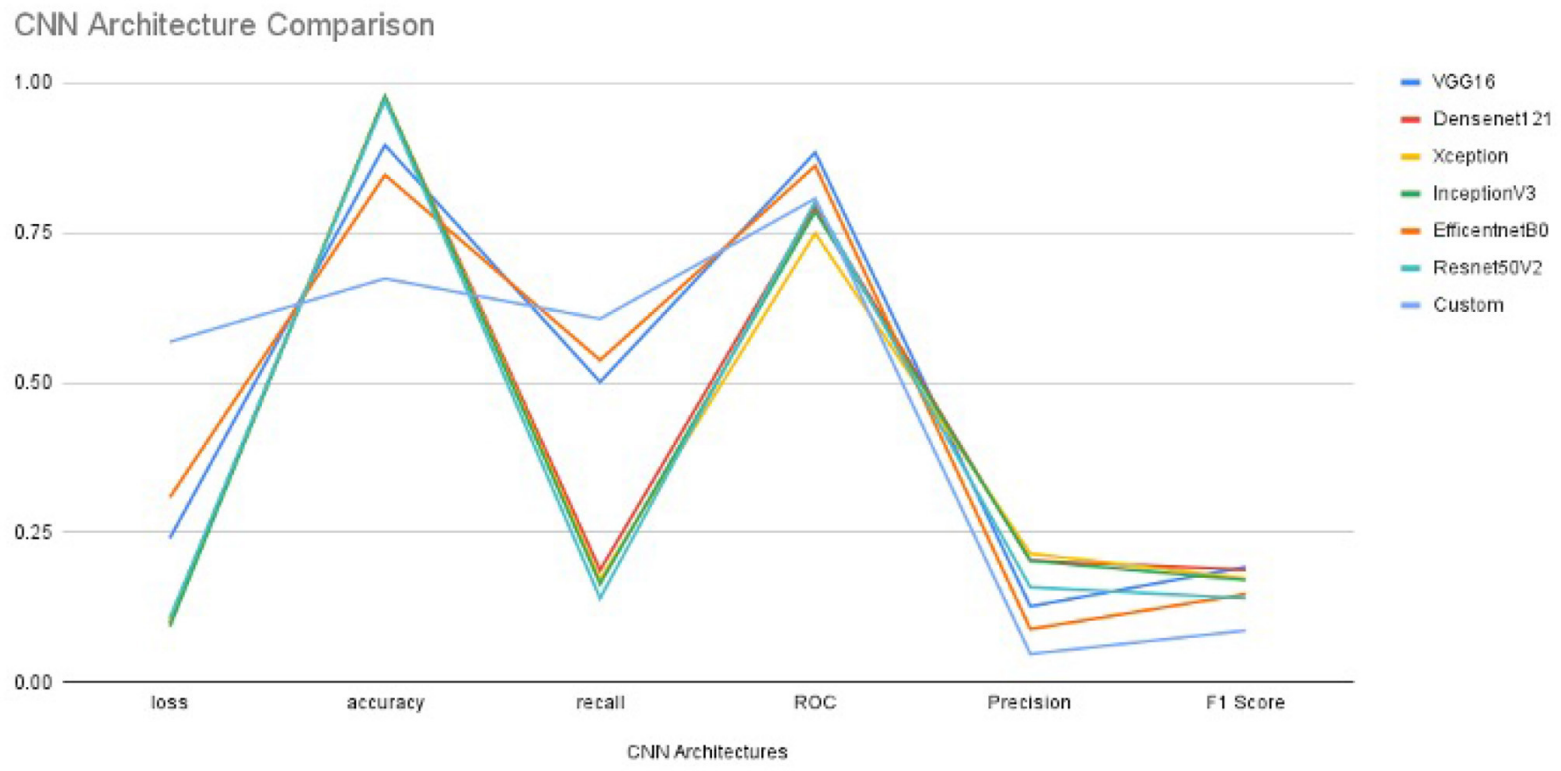
The Performance metrics of CNN architectures with one epoch.

The values of performance metrics of CNN are mentioned in Table 1. It includes the best results of different architecture of CNN. Each CNN's architecture ran under initialized parameters; then, the input parameters were tuned in order to get a better value of performance metric. The tuned parameters include changing the learning rate after every iteration (epoch): changing many optimizers, adding an appropriate number of layers in each base architecture to get a better learning rate, early stopped to get better performance metrics, and changing the dataset split ratio among training, test, and validation dataset. The best values are mentioned in the table after observing several attempts under different configurations of the architectures. Table 1 and Figure 3 illustrate that value of recall and ROC of VGG16 and EfficentNetB0 is better in comparison to the rest of the CNN architectures. In contrast, the F1 Score of VGG16 and DenseNet21 is significantly higher than the other CNN architectures.

**Table 1:** Performance metrics values of convolutional neural network (CNN) with one epoch.

**CNN architecture**	**Loss**	**Accuracy**	**Recall**	**ROC**	**Precision**	**F1 Score**
**VGG16**	0.2407	0.8958	0.5010	0.8835	0.1270	0.1926
Densenet121	0.1000	0.9758	0.1875	0.7938	0.2031	0.1880
Xception	0.1002	0.9766	0.1719	0.7488	0.2148	0.1734
InceptionV3	0.0938	0.9768	0.1646	0.7853	0.2031	0.1709
EfficentNetB0	0.3087	0.8459	0.5375	0.8612	0.0891	0.1475
Resnet50V2	0.1078	0.9705	0.1406	0.8015	0.1589	0.1406
Custom	0.5678	0.6733	0.6062	0.8066	0.0477	0.0867

The final results are calculated using the CNN model. The Keras function (model.evaluate()) tests the model on new data after getting new weights from training. The reason for the low score of the precision and the high score of the accuracy is the data imbalance. The precision is calculated with the number of true positives divided by true positives and false positives. It is favorable for the model to guess that all images are benign or negative as a large portion of images is benign. Since such a large portion of images are benign, it is more likely that an optimistic guess is incorrect, yet we still need to guess positives without giving everything as benign. Thus, the accuracy is very high, but the precision is relatively low.

Furthermore, the accuracy value is significantly closer to each other except for EfficentNetB0 and customized architectures. Since our dataset is heavily imbalanced (based on our observation) and the F1 score does not rely on the hits, we considered the F1 score as the best performance metric for CNN architectures in our experiment. Also, the F1 score is better since it is dependent on the values of precision and recall.

**Figure 4:**
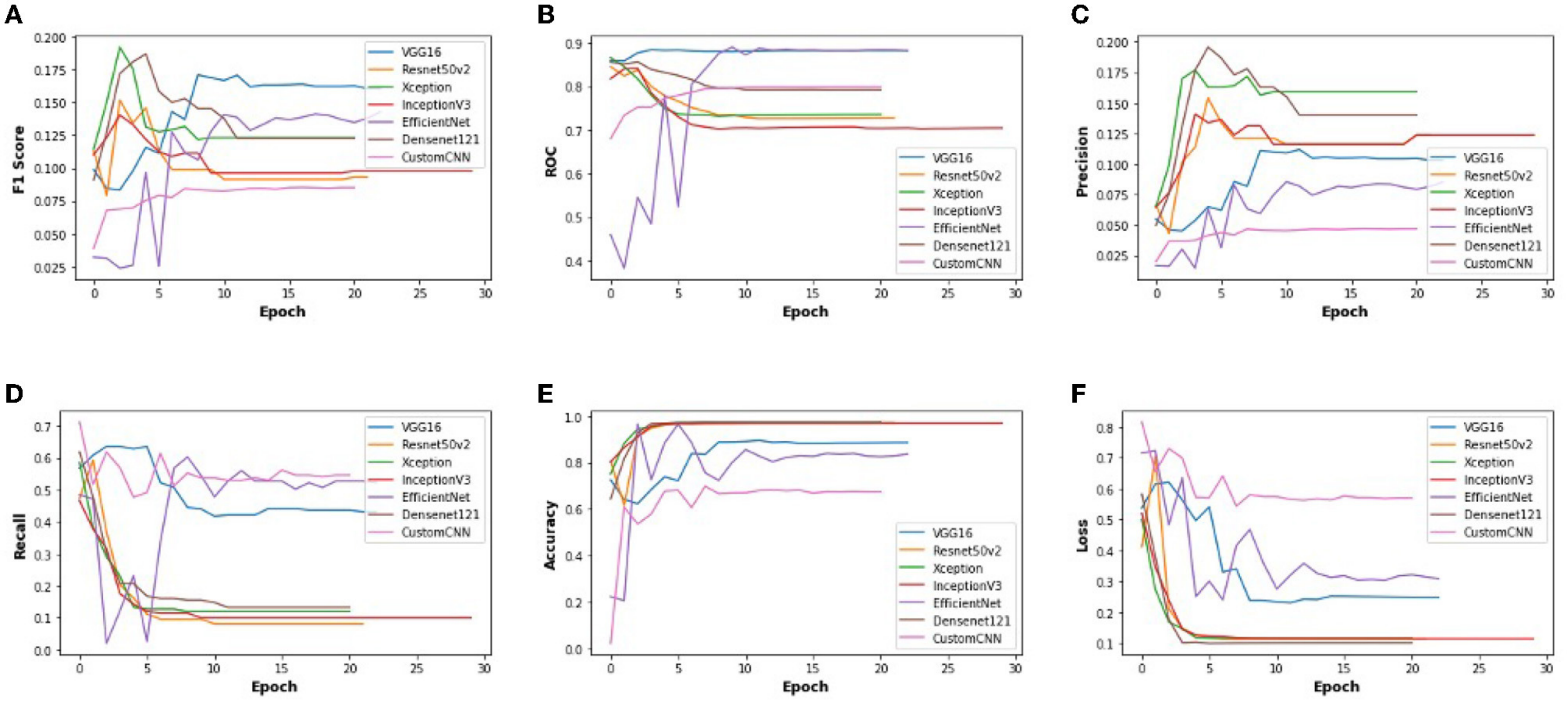
The performance metrics of different architecture of CNN with the variable number of epochs **(A)** F1 score, **(B)** ROC, **(C)** Precision, **(D)** Recall, **(E)** Accuracy, and **(F)** Loss.

In order to get optimal values of performance metrics for different CNN architectures, we ran our model for considerable numbers of epochs. Figure 4 shows the values of performance metrics separately for different architectures. The final evaluation metric is slightly different from what it is for the validation data. However, the values of performance metrics are not significantly different.

### 4.2. Outcome of RNN

The values of performance metrics of RNN are mentioned in Table 2. It includes the best results of the four architectures of RNN. The overall approach was similar to CNN, but we handled the image data matrix differently. First, the image matrix had to be resized, reshaped, and normalized. Also, because the Keras API could not take multiple color channels (R, G, B) at the same time, the images had to be converted to black and white images. This way, we had an only one-color channel for each image, and the overall dimension of the input data was limited.

**Table 2:** Performance metrics values of recurrent neural network (RNN) with one epoch.

**RNN architecture**	**Loss**	**Accuracy**	**Recall**	**ROC**	**Precision**	**F1 Score**
Bidirectional	0.6698	0.8319	0.1976	0.6887	0.0526	0.0778
LSTM	0.5918	0.5300	0.4300	0.7444	0.0389	0.0703
GRU	0.6171	0.6872	0.2874	0.7101	0.0361	0.0625
Simple RNN	0.6751	0.9212	0.0641	0.6045	0.0351	0.0413

**Figure 5:**
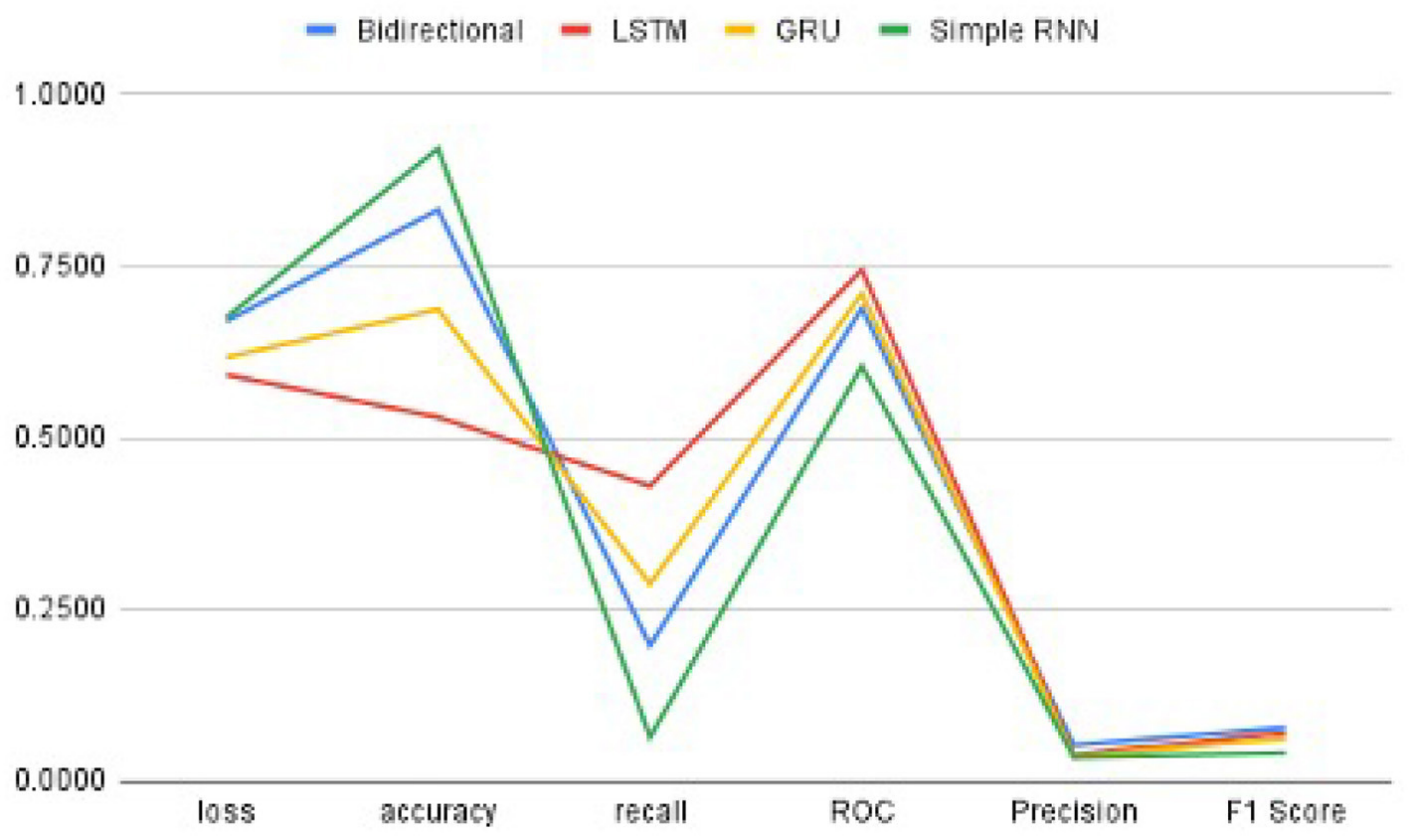
The Performance metrics of RNN architectures with one epoch.

Moreover, RNN works better with time-series data. To utilize these images, we modeled the image data as time series by leveraging the image height and width as time and feature, respectively. Also, the learning rate was tuned such that it starts at a slightly higher value but is gradually decreased after every other epoch. We used SGD optimizers for all the architecture, giving the best overall performance. The best values are mentioned in the table after observing several attempts under different configurations of the four architectures. Figure 5 and Table 2 illustrate that the value of the bi-directional architecture worked the best in terms of its F1 score and accuracy together. However, the other metrics values are not significantly different. Since our dataset is heavily imbalanced (based on our observation) and the F1 score does not rely on the hits, we considered the F1 score as the best performance metric for these architectures in our experiment. Also, the F1 score is better since it is dependent on the values of precision and recall.

**Figure 6:**
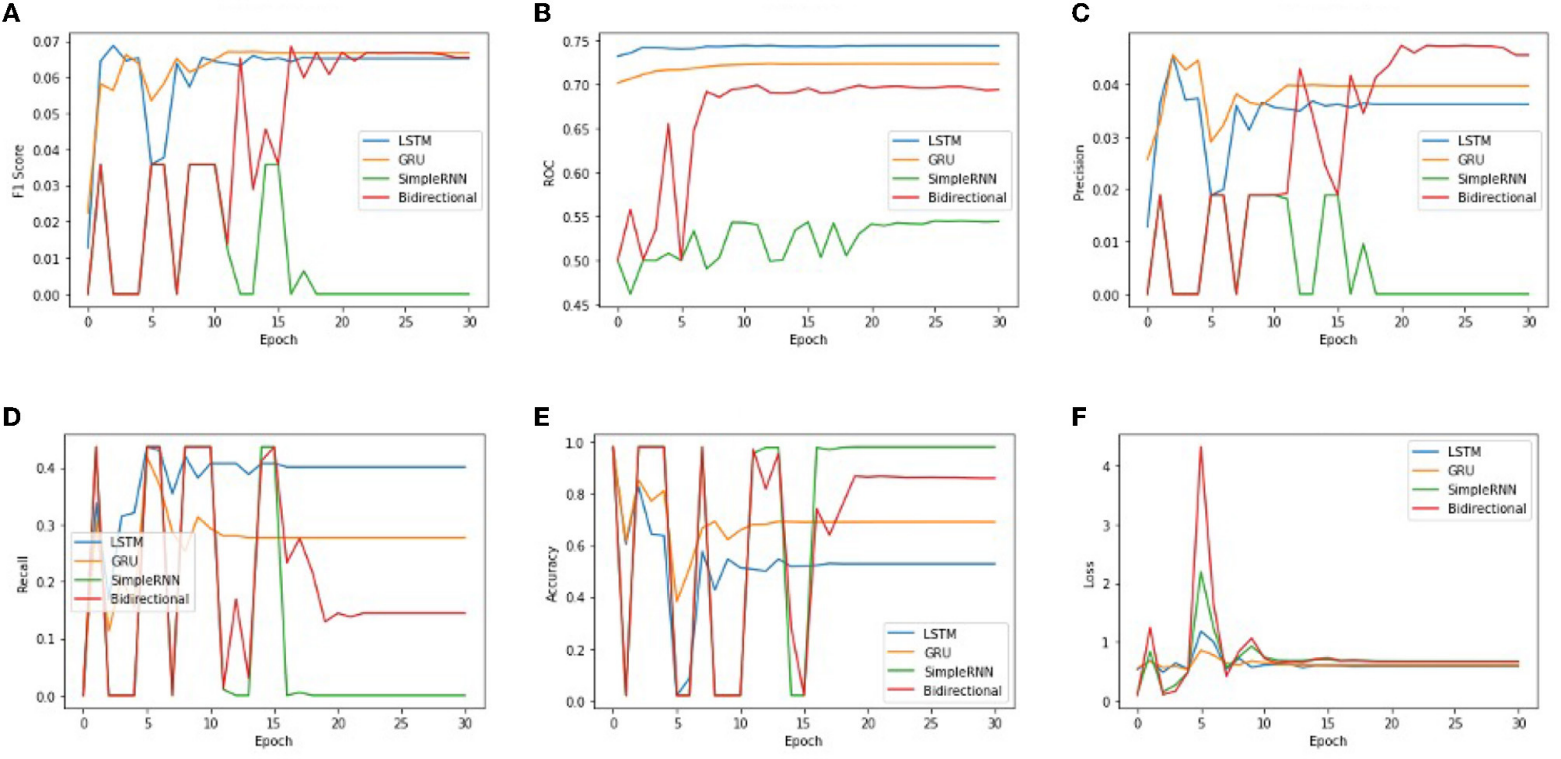
The performance metrics of different architecture of RNN with the variable number of epochs **(A)** F1 score, **(B)** ROC, **(C)** Precision, **(D)** Recall, **(E)** Accuracy, and **(F)** Loss.

In order to get optimal values of performance metrics for four RNN architectures, we ran our model for considerable numbers of epochs. Figure 6 shows the values of performance metrics separately for different architectures. The final evaluation metric is slightly different from what it is for the validation data. However, the values of performance metrics are not significantly different.

### 4.3. Outcome of XG-Boost

**Figure 7:**
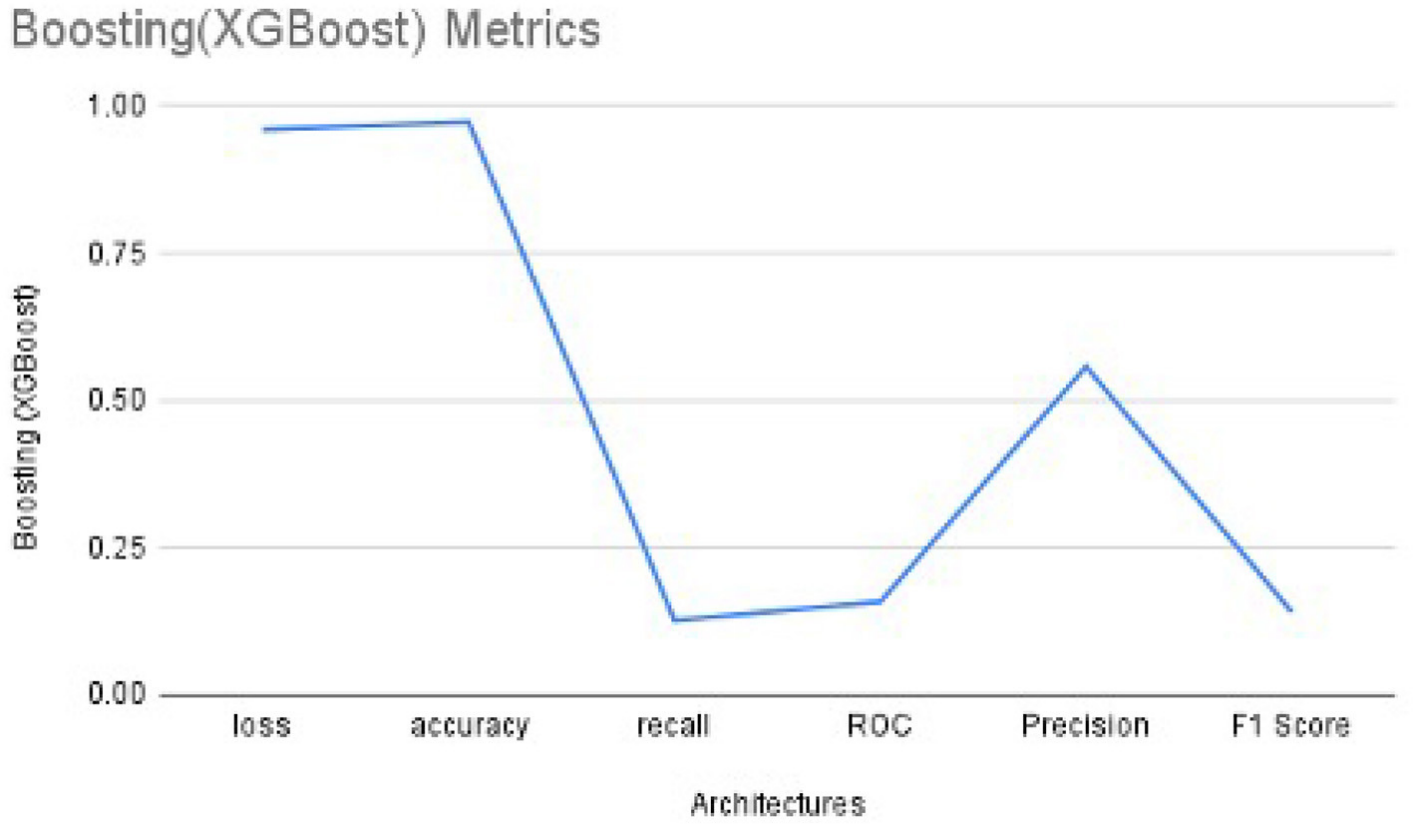
The Performance metrics of XG-Boost with one epoch.

**Figure 8:**
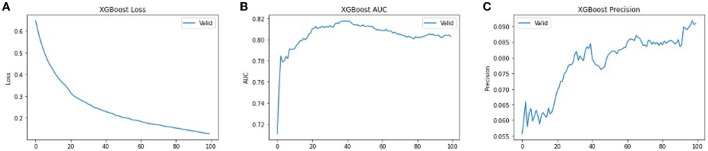
The performance metrics of different architecture of XG-Boost with variable number of epochs **(A)** Loss, **(B)** ROC, and **(C)** Precision.

We used XG-Boost ensemble learning to use the pre-trained VGG16 model as a feature extractor. The extracted features were used as input to the XG-Boost model, with earlier initial parameters. The values of performance metrics of XG-Boost are mentioned in Table 3 and are illustrated in Figures 7, 8. It includes the best result under XG-Boost. It ran under initialized parameters; then, the input parameters were tuned in order to get a better value of the performance metric. The tuned parameters include changing the learning rate (eta) after every iteration (epoch), changing many optimizers (n_estimators), early stopped to get better performance metrics, and altering the dataset split ratio among training tests and validation datasets. The best values are mentioned in the table after observing for several attempts under different configurations. Figures 7, 8 illustrates that value of accuracy is approximately 97%, although the value of the F1 score is lower.

**Table 3:** Performance metrics values of XG-Boost with one epoch.

**Boosting**	**Loss**	**Accuracy**	**Recall**	**ROC**	**Precision**	**F1 Score**
XGBoost	0.9592	0.9722	0.1260	0.1578	0.5568	0.1401

In order to get optimal values of the performance metric of XG-Boost, we ran our model for considerable numbers of epochs. Figures 8A,B shows that the values of performance metrics are converging. The final evaluation metric is slightly different from what it is for the validation data. However, the values of performance metrics are not significantly different.

### 4.4. Comparison of CNN, RNN, and XG-Boost Outcomes

**Figure 9:**
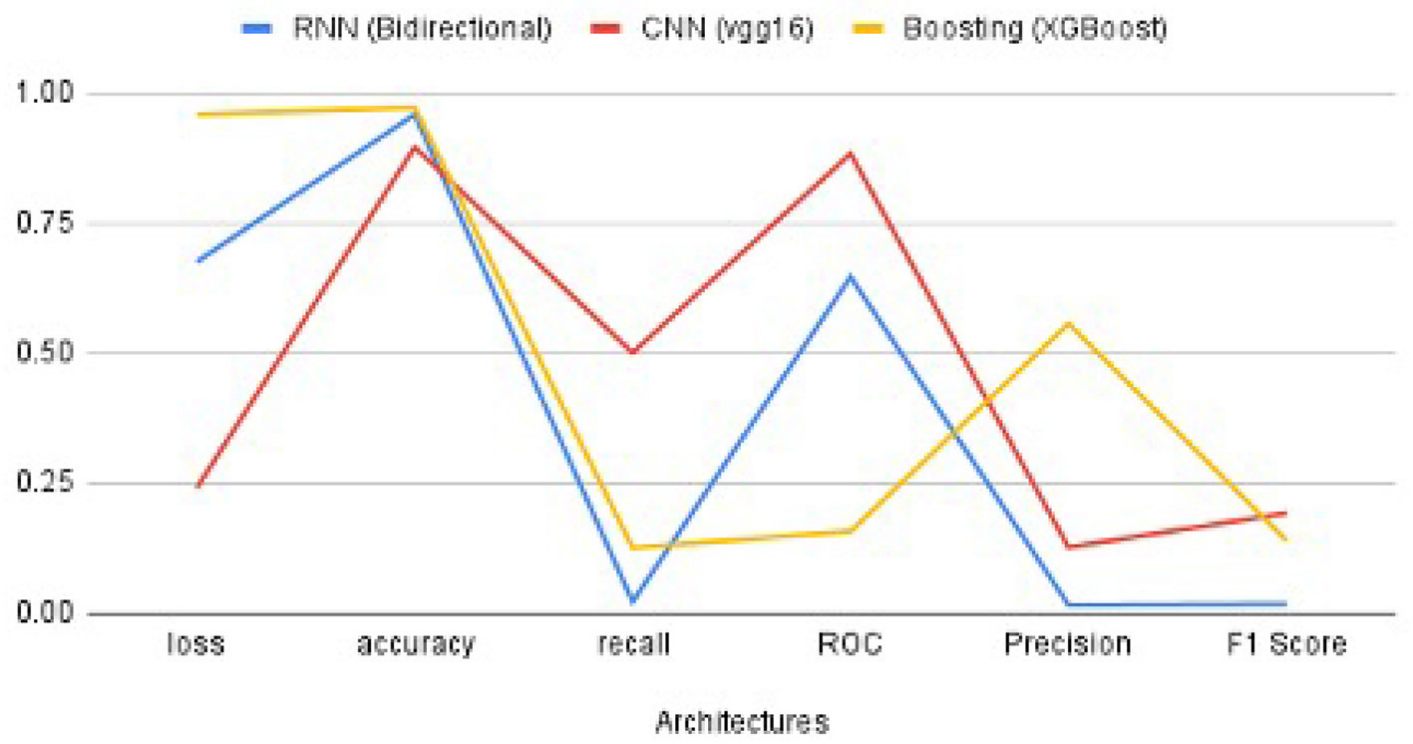
The comparison of performance metrics of CNN, RNN, and XG-Boost.

The performance metrics of CNN, RNN, and XG-Boost are listed in Table 4 and are illustrated in Figure 9. We found that the performance of VGG16 architecture, with an accuracy of 89.6%, is the best architecture (among the other seven architectures) for CNN. Additionally, the RNN's bidirectional architecture is better (accuracy:95.96%) among the other four architectures of RNN. The accuracy of the XG-Boost method is 97.22%.

**Table 4:** Values of best performance metrics of CNN, RNN, and XG-Boost with one epoch.

**Architecture**	**Loss**	**Accuracy**	**Recall**	**ROC**	**Precision**	**F1 Score**
CNN (VGG16)	0.2407	0.8958	0.5010	0.8835	0.1270	0.1926
RNN (Bidirectional)	0.6754	0.9590	0.0224	0.6467	0.0160	0.0182
Boosting (XGBoost)	0.9592	0.9722	0.1260	0.1578	0.5568	0.1401

Based on the values of metrics in Table 4 and shown in Figure 9, CNN's metrics values are much better among the three methods. Most of the performance metrics are higher than RNN and XGBoost (except loss which should be less anyways). One of the reasons for this is the changes required to transform the input data to fit the RNN model. We do lose important features because of this transformation. Also, CNN is more geared for classification problems; as a result, it has better results. XGBoost did get higher Precision and Accuracy, but these metrics do not give the overall picture as the F1 score. While XGBoost generally better, it seems to perform well if there is a limited set of data, which was not the case here.

**Figure 10:**
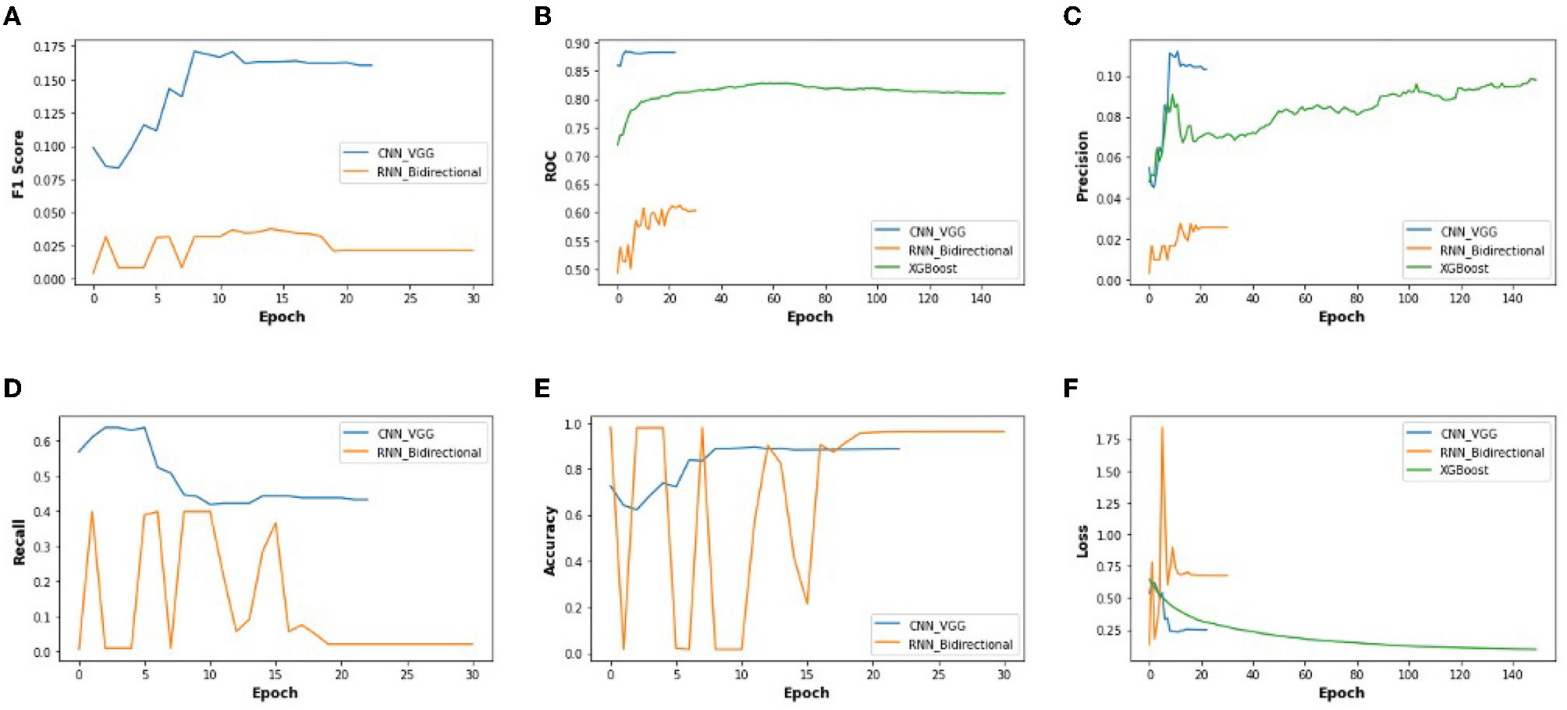
The performance metrics of different architecture of CNN, RNN, and XG-Boost with the variable number of epochs **(A)** F1 score, **(B)** ROC, **(C)** Precision, **(D)** Recall, **(E)** Accuracy, and **(F)** Loss.

Figure 10 illustrates the validation metrics from before, combined for the selected CNN and RNN architectures, and XGBoost. It shows better contrast among the three methods. (XGBoost validation data is only for 3 of these metrics). We also found that the CNN converges in twenty epochs onward, whereas the RNN converges at thirty epochs onward, although XG-Boost took more than one hundered epochs.

Finally, it is noticeable that the CNN, RNN, and XG-Boost achieved much greater accuracy than the dermatologists. The combination of human and deep learning methods' decision to achieve higher accuracy is not limited to the classification of skin cancer of static lesions. Still, it can also be extended to the prospective evaluation of other medical images.

## 5. Conclusion

Based on the experimental outcome of three methods -CNN, RNN, and XG-Boost- of deep neural networks, we found that CNN is generally better than RNN for any architectures used. Within CNN, VGG16 performed the best. CNN with VGG16 seemed to perform better than XG-Boost as well. We would like to continue this process to try our other deep learning algorithms since datasets images are imbalanced and hazy. Moreover, we think there is still some scope for improvements in our runs for CNN, RNN, and XG-Boost. We would like to try out other variations for CNN and further fine-tune the hyperparameters. For RNN, we would like to develop architectures that have many layers, something that we did with CNN using various base models. For XG-Boost, instead of simply using the pre-trained model, we may combine the trained CNN with the XG-Boost classifier.

In order to prevent skin cancer, it has been observed that a typical nevus (moles) is the most decisive risk factor in fair-skinned populations. In particular, the person who has phenotype—pale complexion, blue eyes, and red or fair hair—and high intermittent exposure to solar Ultra Violet A (UVA) radiation are at substantial risk since the UVA penetrates the deeper layers of the skin, which enhance the development of skin cancer, especially if sunburns happen at an early age. Also, the other environmental factors affect a person's UVA exposure, including time of the day and year, latitude, altitude, haze and clouds, ozone, and ground reflection (Cancer-Society, 2021; WHO-Cancer, 2021).

## Data Availability Statement

Publicly available datasets were analyzed in this study. This data can be found at: https://www.kaggle.com/c/siim-isic-melanoma-classification/data.

## Author Contributions

All authors listed have made a substantial, direct, and intellectual contribution to the work and approved it for publication.

## Funding

We gratefully acknowledge the support of the Research Release Time and Gran-in-Aid from the Fairleigh Dickinson University, Teaneck, New Jersey 07666.

## Conflict of Interest

The authors declare that the research was conducted in the absence of any commercial or financial relationships that could be construed as a potential conflict of interest.

## Publisher's Note

All claims expressed in this article are solely those of the authors and do not necessarily represent those of their affiliated organizations, or those of the publisher, the editors and the reviewers. Any product that may be evaluated in this article, or claim that may be made by its manufacturer, is not guaranteed or endorsed by the publisher.
